# The Applications of Ultra-Thin Nanofilm for Aerospace Advanced Manufacturing Technology

**DOI:** 10.3390/nano11123282

**Published:** 2021-12-03

**Authors:** Guibai Xie, Hongwu Bai, Guanghui Miao, Guobao Feng, Jing Yang, Yun He, Xiaojun Li, Yun Li

**Affiliations:** 1 National Key Laboratory of Science and Technology on Space Microwave, China Academy of Space Technology (Xi’an), Xi’an 710100, China; baihw_cc@126.com (H.B.); miao_guanghui@163.com (G.M.); fenggb001@163.com (G.F.); yangjing_215@126.com (J.Y.); hawkinsky@163.com (Y.H.); 2 Hangzhou HTYS Information Technology Co., Ltd., Hangzhou 310024, China; 3 Polytechnic Institute, Zhejiang University, Hangzhou 310015, China

**Keywords:** nanofilm, atomic layer deposition (ALD), secondary electron, titanium nitride, advanced manufacturing

## Abstract

With the development of industrial civilization, advanced manufacturing technology has attracted widespread concern, including in the aerospace industry. In this paper, we report the applications of ultra-thin atomic layer deposition nanofilm in the advanced aerospace manufacturing industry, including aluminum anti-oxidation and secondary electron suppression, which are critical in high-power and miniaturization development. The compact and uniform aluminum oxide film, which is formed by thermal atomic layer deposition (ALD), can prevent the deep surface oxidation of aluminum during storage, avoiding the waste of material and energy in repetitive production. The total secondary electron yield of the C/TiN component nanofilm, deposited through plasma-enhanced atomic layer deposition, decreases 25% compared with an uncoated surface. The suppression of secondary electron emission is of great importance in solving the multipactor for high-power microwave components in space. Moreover, the controllable, ultra-thin uniform composite nanofilm can be deposited directly on the complex surface of devices without any transfer process, which is critical for many different applications. The ALD nanofilm shows potential for promoting system performance and resource consumption in the advanced aerospace manufacturing industry.

## 1. Introduction

The application of aerospace technology is becoming increasingly important due to the rapid development of information and communication technology. The industrial demands promote the rapid growth of aerospace science and technology. The miniaturization and high-frequency development of aerospace microwave components are becoming increasingly important. More advanced technologies need to be adopted to satisfy the demands of better performance and tougher standards of components. Advanced aerospace manufacturing technology is attracting increased attention, and researchers have developed many technologies to achieve better performance, reduced energy and resource consumption, more reliable products, etc. [1,2]. The multipactor, which is induced by secondary electron emission (SEE) under an RF electric field in a vacuum due to excessive energy density, is one problem that needs to be solved for high-power aerospace microwave technology. The multipactor can deteriorate the performance of high-power microwave components in communication satellites [3,4,5]. Several approaches have been developed to suppress the secondary electron yield (SEY), which is critical for the suppression of the multipactor. These approaches can be divided into two kinds: one is the artificial surface roughness, and the other is the intrinsic low-SEY coating. The former takes advantage of the nanostructure of the electron traps, which could confine low-energy electrons and yield a lower surface SEY [6,7,8]. The suppression of the SEY based on a low-SEY coating is more effective and can avoid degrading the electrical performance of the microwave components. Since the intrinsic SEE properties of solid materials are determined by the component element, chemical bonding, and crystalline structure, several appropriate materials are selected to decrease the SEY, including alodine, carbon [9,10,11], graphene [12,13,14], and titanium nitride [15,16,17,18,19].

Atomic layer deposition (ALD) is a unique chemical vapor deposition technology that demonstrates two properties: surface self-saturation and ordinal reaction [20]. It is generally applied in the preparation of functional nanomaterials, such as lithium batteries [21], nanostructures [22,23], optoelectronic devices [24], and nanocatalysis [25]. It is worth pointing out that the ALD nanofilm is compact and has a controllable sub-nanometer thickness. During the thermal evaporation and sputtering process, nanoparticles accumulate continuously to form polycrystal nanofilm, resulting in poor film compactness. In the ALD process, the reaction source precursors chemically absorb on the surface, and atoms are covalently bonded together.

In this paper, we report the applications of ultra-thin ALD nanofilm for the advanced aerospace manufacturing industry. The compact aluminum oxide nanofilm was prepared through thermal ALD. Secondary electron yield was employed to investigate the surface oxidation of aluminum before and after ultra-thin aluminum oxide nanofilm deposition. The result indicated that the ALD nanofilm can prevent oxidation, and the investigation provided a general route for high-quality surface treatment and storage in the advanced aerospace manufacturing industry. The titanium nitride and amorphous carbon were prepared through plasma-enhanced ALD. The secondary electron yield of composite nanofilm decreases by 25% compared with an uncoated sample. The suppression of secondary electron emission is of great importance in solving multipactor for high-power microwave components in space and the electron cloud for large, high-energy particle accelerators. The study provided an important approach to suppress the multipactor and increase the threshold value for the advanced aerospace manufacturing industry. The ultra-thin ALD nanofilms improve the power threshold of high-power microwave components and prevent the oxidation of typical metals, which demonstrates great potential in the advanced aerospace manufacturing industry.

## 2. Materials and Methods

**Figure 1:**
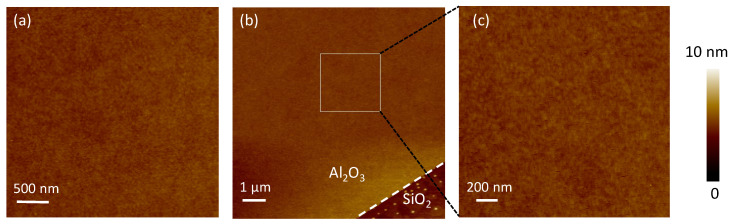
The atomic force microscopy images of SiO_2_ before (**a**) and after (**b**,**c**) Al_2_O_3_ deposition.

In this work, two different ALD modes are adopted: thermal ALD and plasma-enhanced ALD (R-200 Advanced, Picosun, Espoo, Finland). The aluminum oxide nanofilms are prepared through thermal ALD using trimethyl aluminum (TMA, Sigma-Aldrich, Shanghai, China) as precursors [26]. The oxidizer is water. In order to remove the natural oxide layer, the aluminum sample (99.99% purity, HFKJ, Hefei, China) was cleaned by argon ion with the energy of 800 eV in a vacuum and immediately stored in nitrogen. After loading the samples into the ALD reaction chamber, a flow of high-purity N_2_ was used throughout the deposition process as a purge gas. The waiting time was 90 s to remove the excess precursor. The deposition temperature was 130 °C. An atomic force microscope (AFM, Dimension Edge, Bruker, Billerica, MA, USA) was adopted to characterize the film deposited on SiO_2_ (HFKJ, Hefei, China), which was prepared under the same conditions. Figure 1 shows the AFM before and after the ALD process. In ALD, the selective edge deposition effect was adopted to characterize the thickness of the nanofilm [26]. In Figure 1b, the thickness of aluminum oxide film was about 3 nm for 40 cycles. The deposition rate was 0.08 nm in each cycle. For ALD technology, high-quality nanofilm shows high uniformity and density. It is the key to keeping the material off the oxygen and water vapor. The roughness of the nanofilm was about 0.21 nm, which was equivalent to the roughness of the SiO_2_ substrate at 0.17 nm. No pinhole was detected.

**Figure 2:**
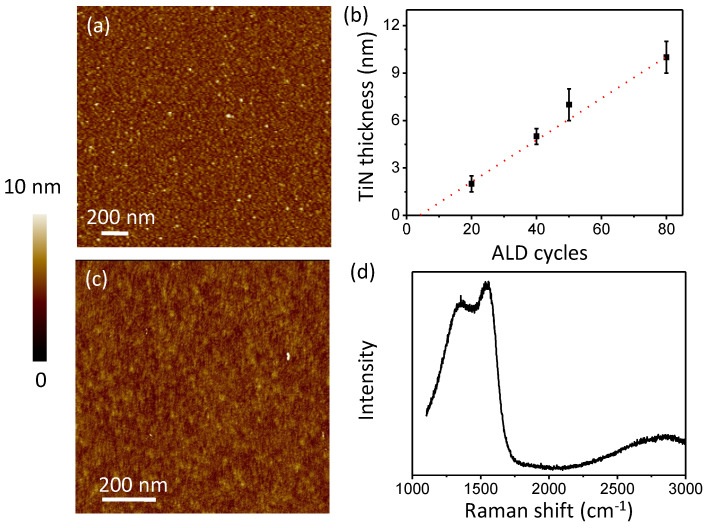
The titanium nitride and amorphous carbon nanofilms. (**a**) AFM image of TiN film on SiO_2_; (**b**) the ALD rate of TiN; (**c**) AFM image of amorphous carbon film on SiO_2_; (**d**) Raman spectra of amorphous carbon film.

The titanium nitride and amorphous carbon nanofilms are prepared through the PEALD process at a low temperature. Tetrakis(dimethylamino)-titanium (TDMAT, Sigma-Aldrich, Shanghai, China) and NH_3_ plasma are selected as the titanium and nitrogen precursors, respectively. Carbon bromide is selected as the carbon precursor. The aluminum alloy with plating silver substrates is loaded via a load-lock chamber. Before the PEALD process, the substrates were annealed at 160 °C for 30 min. In order to avoid the deterioration of the aluminum alloy, we chose a lower deposition temperature. The deposition temperature was 160 °C for both TiN and amorphous carbon. After the deposition process, the samples were transferred to the load-lock chamber to cool to the ambient temperature. An AFM was adopted to characterize the film deposited on SiO_2_, which was prepared under the same condition in Figure 2. The roughness of TiN was about 0.39 nm for samples of 40 cycles and 80 cycles. The deposition rate was about 0.12 nm per cycle for TiN. The roughness of amorphous carbon was about 0.30 nm for samples of 20 cycles. The film thickness was controlled by the reaction cycles in the deposition process.

**Figure 3:**
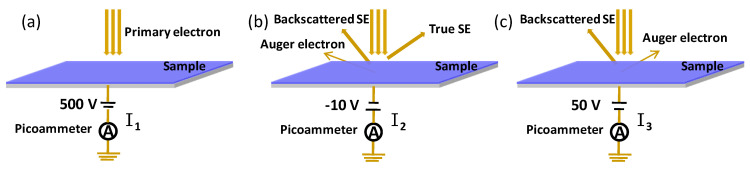
The schematic representation of the experimental system for SEY measurement. (**a**) The sample current I_1_ represents primary currents I_PE_; (**b**) the sample current I_2_ represents I_PE_−I_tot SE_; (**c**) the sample current I_3_ represents I_PE_−I_BSE_−I_AE_.

When electrons with a certain energy are injected into solid materials, electrons can be ejected from the surface. The process is called secondary electron emission (SEE), and the ejected electrons are called secondary electrons (SEs). The ratio of the SE to the injected primary electron is called the secondary electron yield (SEY), which is the key parameter to characterize the SEE property. The secondary electron generated in several nanometers on the surface can escape into the vacuum. The secondary electron yield depends on the elements, crystal structure, and morphology of the material. The SEY is sensitive to surface oxidation, and the oxidation can be deduced by the SEY of the surface. In our studies, the SEY was adopted to characterize the oxidation of aluminum, and the decrease of the SEY provides the key approach to suppress the multipactor in high-power microwave components. The measurement of the SEY was performed in a high-vacuum chamber with a base vacuum pressure of 1.6 × 10^−5^ Pa. The schematic representation of the experimental system for SEY measurement is shown in Figure 3.

The secondary electron yield was studied by the conventional sample–current method, and the electron beam current was confirmed by the Faraday cup [27,28]. To avoid charging problems, the electron dose during the measurement was set below 1 × 10^−8^ C/mm^2^ with irradiated areas of about 0.1 mm^2^. All of the primary electrons were collected in the sample when biased at +500 V in Figure 3a. It is worth pointing out that the BSEs with over 500 eV might escape from the sample when the energy of the injected electron was higher than 500 eV. The measured primary current was a little smaller than the real current. The SEY was larger than that it should be. When negatively biased, the true SEs and backscattered SEs escaped from the sample and were ejected into the vacuum. Four different negative voltages were tested to improve the precision of I_2,_ including 0, −5 V, −10 V, and −20 V. The results showed that when biased at −5 V, −10 V, and −20 V, the TSEs and BSEs were completely ejected into the vacuum. However, when biased at 0 V, some surface SEs cannot escape from the surface, which leads to a decrease in the SEY. The negative bias was set as −10 V in Figure 3b. When 50 V were applied, the TSEs were pulled back into the sample, and the BSEs escaped into the vacuum in Figure 3c. The sample currents I_1_, I_2_, and I_3_ represent the primary currents I_PE_, (I_PE_−I_tot SE_), and (I_PE_−I_BSE_−I_AE_), respectively. The primary electron current I_1_ is also confirmed by the Faraday cup.

## 3. Results and Discussion

**Figure 4:**
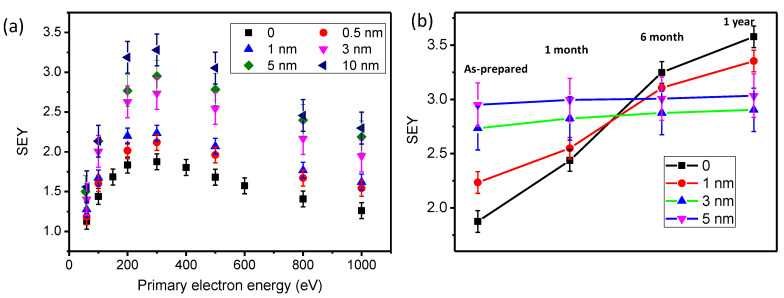
The SEY measurement of aluminum with different ALD processes. (**a**) The SEY as a function of primary electron energy with different thicknesses; (**b**) the change of SEY_max_ with time.

The SEY of aluminum was measured before and after ALD in Figure 4. Samples with different thicknesses were investigated. The SEY of aluminum without ALD increased with the primary energy and reached its peak at 300 eV. The maximum SEY was 1.87 ± 0.1. To prevent the oxidation of aluminum before the ALD process, the samples were stored in a vacuum of the load-lock chamber. When the reaction condition was stable, the aluminum was transferred into the reaction chamber, and the ALD process was performed. Each of the samples with different thicknesses was completed by a separate deposition. The SEY_max_ increased to 2.12 ± 0.1, 2.23 ± 0.1, 2.73 ± 0.2, 2.95 ± 0.2, and 3.28 ± 0.2 for samples with a 0.5 nm-coating, 1 nm-coating, 3 nm-coating, 5 nm-coating, and 10 nm-coating, respectively. The SEY_max_ increases with the oxide layer, and the qualitative analysis of surface oxidation can be obtained through the SEY measurement. Compared with the uncoated sample, there was a considerable increase for the SEY of aluminum with a 0.5 nm coating. Oxidation cannot be completely avoided before the ALD process. Thus, there was an unavoidable, additional, thin oxide layer on the sample. The SEY measurement was unstable due to the charge accumulation for samples with a much thicker oxide coating. However, the SEY_max_ of naturally oxidized aluminum could be much larger than the ALD sample under a stable electric current measurement. We deduced that the oxidation film of natural oxidation was incomplete and contained some conductive channels.

After the ALD process, samples with different coatings were stored in a drying cabinet at room temperature. The SEY_max_ was investigated after 1 month, 6 months, and 1 year. The SEY of aluminum without the ALD nanofilm increased rapidly. The naturally oxidized layer was noncompact, and the existing oxide layer could not prevent the inner material from oxidation. When 1 nm aluminum oxide was deposited, the increase of the SEY_max_ was slower than the naked sample. When the thickness of the ALD nanofilm increased to 3–5 nm, there was little change in the SEY_max_. The SEY_max_ of the sample with the 3 nm coating was about 2.9 after 1 year, which was smaller than that of the 5 nm coating. The thickness of the oxide layer after 1 year of storage could be smaller than 5 nm, which indicated that the compact and uniform nanofilm could prevent the oxidation of aluminum.

**Figure 5:**
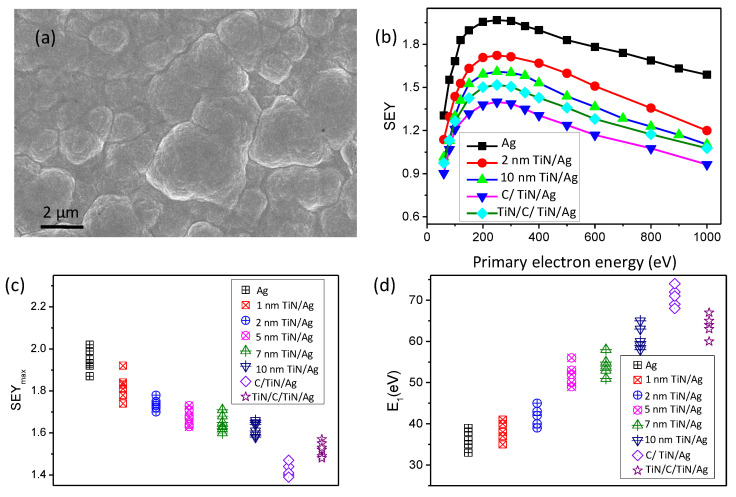
The suppression of secondary electron emission of the ALD composite nanofilm. (**a**) The scanning electron microscope image of a sample after the ALD process; (**b**) the SEY curve as a function of primary electron energy with different nanofilms; (**c**) the SEY_max_ of silver surface with different nanofilm; (**d**) the E_1_ of silver surface with different nanofilm.

The aluminum alloy sample with 6 µm plating silver was annealed in nitrogen before the ALD process. The thickness of the TiN nanofilm was controlled by the deposition cycles. The scanning electron microscope image showed the surface topography after 10 nm TiN nanofilm deposition in Figure 5a. The homogeneity of the ALD film was much better than the plating silver film. Although the conductivity of TiN is better than most low-SEY coatings, it is still worse than that of silver since the skin-depth of high-frequency RF devices is small, especially for the millimeter device. Excessive TiN film can increase the insertion loss. It is important to study the quantitative relationship of the SEY on the film thickness of the outermost TiN. To obtain the optimum thickness of titanium nitride, dozens of samples with different TiN thicknesses were investigated in Figure 5b. In Figure 5c, the SEY_max_ decreased to 1.81 ± 0.05, 1.74 ± 0.05, 1.68 ± 0.05, 1.65 ± 0.05, and 1.64 ± 0.05 for 1 nm, 2 nm, 5 nm, 7 nm, and 10 nm coating samples, respectively. Compared with the naked silver surface, the SEY decreased sharply after the first 2 nm TiN coating. When the thickness increased to 5 nm, the SEY decreased slowly with the increase of the TiN nanofilm. After the 10 nm deposition, the SEY remained almost unchanged with the increase of the TiN nanofilm. The SEY uniformity of the TiN film was studied. Several different positions on the 10 nm TiN sample were selected. The SEY_max_ was 1.64, 1.61, 1.63, 1.62, and 1.63. Then, the SEY of five samples was measured, and the SEY_max_ was 1.64 ± 0.05. The result showed good uniformity. There are two key parameters to determine the multipactor threshold: SEY_max_ and E_1_. The parameter E_1_ is the energy of the incident electron when the SEY reaches 1 for the first time with the increase of the incident electron energy. In low energy, the SEY increases linearly with the incident energy in Figure 5d. The E_1_ can be measured or fitted. The result demonstrated that E_1_ increased with the TiN deposition. E_1_ of the 10 nm coating sample was larger than 5 nm, which indicated the 10 nm TiN nanofilm was the optimum thickness for multipactor suppression of high-power microwave components.

The SEY of carbon materials, such as graphite, is much smaller than that of TiN. However, it is a challenge to prepare high quality, controllable carbon nanofilm on an aluminum alloy surface. In our experiment, the plasma-enhanced ALD can reduce the temperature for a reaction compared with the traditional thermal reaction. Carbon nanofilm was deposited on the surface after 10 nm TiN deposition. Ten cycles of carbon were processed, and the thickness was about 1 nm. The SEY changed greatly after carbon nanofilm deposition, and the SEY_max_ decreased to about 1.41. Moreover, the parameter E_1_ increased to 70 eV. According to the testing experience, the ultra-thin film can increase about 2–3 times the discharge threshold. When 1 nm of TiN was deposited after the carbon, the SEY_max_ would increase, and E_1_ would decrease, which caused damage to the high-power technology. Considering that the TiN nanofilm can also be used as an anti-radiation coating, the ALD composite nanofilm of TiN and carbon shows great potential in high-power spacecrafts. It is necessary to deposit carbon nanofilm on the first top layer.

**Figure 6:**
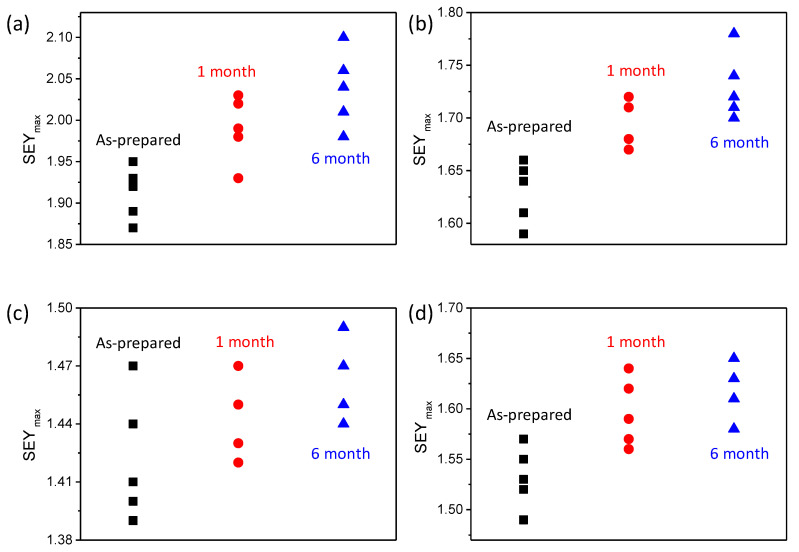
The SEY_max_ of different samples with ALD nanofilm at different times. (**a**) The SEY_max_ of aluminum alloy with plating silver; (**b**) the SEY_max_ of samples with 10 nm TiN on silver; (**c**) the SEY_max_ of samples with 1 nm C/10 nm TiN on silver; (**d**) the SEY_max_ of samples with 1 nm TiN/1 nm C/10 nm TiN on silver.

The stability of the SEY with the ALD nanofilm is very important for engineering applications. The SEY of samples with an ALD nanofilm was studied at different times. The samples were stored in the drying cabinet before the SEY measurement. The SEY_max_ of naked planting silver increased to 2.0 ± 0.05 and 2.05 ± 0.05 after 1 month and 6 months, respectively, as shown in Figure 6a. After 10 nm TiN deposition, the SEY_max_ increased to 1.7 ± 0.05 and 1.72 ± 0.05 from 1.64 ± 0.05. The stability of the SEY after TiN deposition was slightly improved. However, the SEY_max_ of the TiN film increased to 1.95 ± 0.05 due to the absorption of oxygen and water after 6 months in the air. In Figure 6c, the measurement showed a small change of the SEY for the carbon/TiN composite nanofilm sample. Compared with TiN nanofilm, the ALD carbon nanofilm was inactive. When the TiN was deposited on carbon, the SEY of the surface and change increased. The results demonstrated the high-stability of the SEY after ALD composite C/TiN nanofilm coating.

## 4. Conclusions

In this work, we reported on the applications of ultra-thin ALD nanofilm in the aerospace industry. The compact aluminum oxide nanofilms and C/TiN composite nanofilms with controllable thicknesses were formed. The SEY measurement was adopted to study the oxidation of aluminum. The result showed that compact aluminum oxide film could prevent oxidation and maintain the high quality of aluminum during storage. The C/TiN composite nanofilms were formed through plasma-enhanced ALD and could suppress the SEY of silver. The suppression of secondary electron emission was of great importance in solving the multipactor for high-power microwave components in space. The suppression effect was stable in dry air. Moreover, the controllable, ultra-thin, uniform composite titanium nitride film can be formed directly on the complex surfaces of devices without any transfer process, which is critical for many different applications.

## Data Availability

The datasets generated during the current work are available from corresponding author on reasonable request.
